# Improved detection of air-filled lesions using computed tomography in dogs with recurrent spontaneous pneumothorax through reduction of pulmonary atelectasis via positive pressure ventilation

**DOI:** 10.3389/fvets.2024.1325211

**Published:** 2024-01-24

**Authors:** Atsushi Toshima, Caroline V. Fulkerson, Yumiko Kagawa, Masahiro Murakami

**Affiliations:** ^1^Japan Small Animal Medical Center, Saitama, Japan; ^2^Department of Veterinary Clinical Sciences, College of Veterinary Medicine, Purdue University, West Lafayette, IN, United States; ^3^North Lab, Sapporo, Japan

**Keywords:** air-filled lesion, bulla, bleb, lateral thoracotomy, median thoracotomy

## Abstract

**Introduction:**

Spontaneous pneumothorax in dogs is predominantly caused by the rupture of air-filled lesions, such as bullae or blebs. The efficacy of Computed Tomography (CT) in detecting these lesions has been deemed limited due to its reportedly low sensitivity. This retrospective, cross-sectional study investigates the utility of CT in eight dogs diagnosed with recurrent pneumothorax, all of which had surgical confirmation of the cause of the pneumothorax.

**Materials and methods:**

Thoracic radiographs were obtained before and the day following the CT studies. Initially, a CT study was conducted without positive pressure ventilation (pre-PPV CT). Subsequent CT studies were performed post-evacuation of pneumothorax and with positive pressure ventilation of 15 cmH2O until lung atelectasis was resolved (post-PPV CT). The pre-PPV CT and post-PPV CT images were anonymized and reviewed by two board-certified radiologists. The presence and morphology of air-filled lesions were evaluated on all images. Surgical findings were recorded and compared to the CT findings.

**Results:**

Air-filled lesions were detected in 5 out of 8 dogs in the pre-PPV CT studies and in all 8 dogs in the post-PPV CT studies. The CT findings of air-filled lesions were consistent with surgical findings. None of the dogs showed increased severity of pneumothorax in radiographs taken the day following the CT studies.

**Discussions:**

The study concludes that the resolution of lung atelectasis by evacuation of pneumothorax and positive pressure ventilation during CT studies is feasible and enhances the detection of air-filled lesions in dogs with recurrent spontaneous pneumothorax. This could potentially aid in improving surgical planning.

## 1 Introduction

Pneumothorax is characterized by the pathological accumulation of air within the pleural space, leading to secondary pulmonary atelectasis. This condition can be categorized into traumatic, spontaneous, or iatrogenic forms (1). Spontaneous pneumothorax, which is a closed pneumothorax and occurs without iatrogenic or traumatic etiologies, has been associated with various causes in dogs. These include air-filled lesions (such as pulmonary bullae or blebs), bullous or lobar emphysema, bacterial pneumonia, pulmonary abscesses, dirofilariasis, and neoplasia (1–11). Among these, the rupture of air-filled lesions (bullae or blebs) has been identified as the most common cause of spontaneous pneumothorax in dogs (12–15).

Surgical resection of the air-filled lesion is recommended for the treatment of spontaneous pneumothorax in dogs (16, 17). Among the various surgical approaches, intercostal/lateral thoracotomy is favored due to its minimally invasive nature and reduced risk of complications, compared to median sternotomy (18, 19). Precise preoperative detection and assessment of these air-filled lesions are crucial for successful surgical planning, as they inform the choice of the most appropriate surgical approach based on the location and quantity of the air-filled lesions.

Computed tomography (CT) is regarded as a superior modality for detecting pulmonary air-filled lesions in dogs with spontaneous pneumothorax when compared to thoracic radiography (20). Nevertheless, the utility of CT remains somewhat restricted due to its low sensitivity in detecting air-filled lesions (13, 20). This low sensitivity of air-filled lesion detection is considered to be due to presence of pulmonary atelectasis caused by pneumothorax or ruptured air-filled lesion itself, since the reduction of pneumothorax is known to improve detection of air-filled lesion in dogs with spontaneous pneumothorax (21). Evacuation of pneumothorax before the CT study is known to help improvement of air-filled lesion detection, however it is difficult to completely resolve atelectasis which remain limiting the detection of air-filled lesion. Although additional positive pressure ventilation has been shown to improve the visibility of air-filled lesions in both humans and dogs, it carries the potential risk of exacerbating air leakage from the air-filled lesion itself (22, 23).

Thus, the purpose of this study are (1) to determine if evacuation of pneumothorax and concurrent positive pressure ventilation improve the detection rate of air-filled lesions, and (2) to assess the potential risks associated with positive pressure ventilation in canine patients presenting with spontaneous pneumothorax. We hypothesized that the application of PPV to CT scans of canine patients with spontaneous pneumothorax would improve the detection of air-filled lesions without worsening the underlying condition. Specifically, we postulated that the integration of PPV with thoracic tube placement to resolve pulmonary atelectasis would facilitate more accurate identification of air-filled lesions. This, in turn, could aid in surgical planning without leading to an increase in pneumothorax severe enough to require immediate surgical intervention. This approach aims to provide a more reliable and safe method of managing spontaneous pneumothorax in dogs.

## 2 Materials and methods

### 2.1 Selection and description of subjects

This was retrospective, cross-sectional study. Medical record search was conducted between April 2018 and February 2022, including the dogs with recurrent pneumothorax with no radiographic or clinical evidence of trauma. The dogs without surgical confirmation of the cause of spontaneous pneumothorax were excluded. Due to the retrospective study design, institutional animal care approval was not required. However, the use of medical record data was approved by the hospital owner/director and animal owners.

#### 2.1.1 Data recording and analysis

All thoracic CT scans were performed using an 80-slice multi-detector CT scanner (Aquilion PRIME; Canon, Tokyo, Japan) by a veterinarian with 14 years of radiology experience (AT). Each dog was positioned in sternal recumbency under general anesthesia, and manual ventilation was performed using a reservoir bag. In all dogs, apnea was induced immediately prior to each CT study. We ensured that all scans were obtained during the expiratory phase when the patients were in a state of apnea. Standard CT parameters included a helical scan mode, pitch of 1.388, 0.5 s rotation time, 120 kVp, 350 mA, and 0.5 mm slice thickness. Initially, a CT study without positive pressure ventilation (pre-PPV CT) was performed. Evacuation of pneumothorax and placement of thoracic tube before pre-PPV CT was recorded. Following air evacuation from the pleural cavity via thoracic tube or thoracocentesis, a PPV CT scan was performed with positive pressure ventilation. Manual compression of a reservoir bag was used for approximately 30 to 60 s, to achieve each end-inspiration pressure of 15 to 20 cmH2O for PPV. Breath holding at a positive end-expiratory pressure of 15 cmH2O was maintained throughout PPV CT image acquisition by anesthesia machine (RO-45Va, ACOMA Medical Industry Co., Ltd., Tokyo, Japan), with no personnel remained in the CT room during the actual CT scanning process. If lung atelectasis persisted in the PPV CT, PPV was again applied for 2–5 min using manual compression of the reservoir bag at end-inspiration pressures of 15–20 cmH2O, followed by a PPV CT scan with breath holding (positive end-expiratory pressure of 15 cmH2O). Pre-PPV CT and the last PPV CT (post-PPV) study performed were used for further analysis.

#### 2.1.2 Image analysis of air-filled lesions

Both the pre-and post-PPV CT of the thorax, in the form of digital files, were anonymized and randomized prior to evaluation. Two ACVR board-certified radiologists (MM and CF) assessed the CT images via consensus using an image viewing workstation (Osirix MD: Pixmeo, Geneva, Switzerland).

The presence of air-filled lesions and the following CT criteria for air-filled lesions were evaluated if present; (i) number, (ii) size (maximal diameter; mm), (iii) location (lung lobe), (iv) peripheral or central, (v) shape (rounded or irregular). The severity of the pneumothorax was subjectively evaluated for each pleural cavity (right or left) using a classification system: mild (amount of gas in each pleural space <25%), moderate (25%–50%), and severe (>50%).

#### 2.1.3 Assessment of potential risks of PPV

Thoracic radiographs were obtained before and the following morning of the CT study and degrees of pneumothorax were evaluated in each dog by a veterinarian with 14 years of experience in radiology (AT). Respiratory rate and the volume of air evacuated between the CT study and the following morning were documented.

#### 2.1.4 Surgical confirmation of air-filled lesions

Surgical reports were reviewed, recorded, and used to compare the location and number of air-filled lesions with CT findings. Histopathological diagnoses were also recorded. A thorough review of all dogs’ medical records was conducted, and any instances of pneumothorax recurrence were documented, if present.

## 3 Results

### 3.1 Study population

Eight dogs met the inclusion criteria. All 8 dogs were purebreds including 2 Golden Retrievers, 2 Shiba Inus, 1 Chihuahua, 1 Standard Poodle, 1 Labrador Retriever, and 1 French Bulldog. The median age was 7.4 years (range, 3 to 12 years).

### 3.2 Thoracic tube placement and thoracocentesis prior and during CT study

Three dogs had a unilateral thoracic tube placement in the right hemithorax prior to the pre-PPV CT scan. Of the eight cases studied, 5 stable dogs without severe respiratory distress or low SpO_2_ levels underwent pre-PPV CT without prior thoracocentesis or tube placement. In all cases, preparations were made for thoracic tube placement at any time during the imaging procedure.

Three additional thoracic tube placements were performed between the pre-PPV CT and post-PPV CT scans, one in the left hemithorax and two in the right hemithorax, for a total of 6 of 8 dogs that received unilateral thoracic tube placement prior to the post-PPV CT scan. In the remaining two dogs that did not receive thoracic tube placement, thoracocentesis was performed on the right hemithorax between the pre-PPV and post-PPV CT scans.

In all 8 cases, gas evacuation was performed unilaterally either by thoracic tube or thoracocentesis, with one procedure performed on the left hemithorax and the remaining 7 procedures performed on the right hemithorax between the pre-PPV and post-PPV CT scans.

### 3.3 Image analysis of air-filled lesions

**Figure 1:**
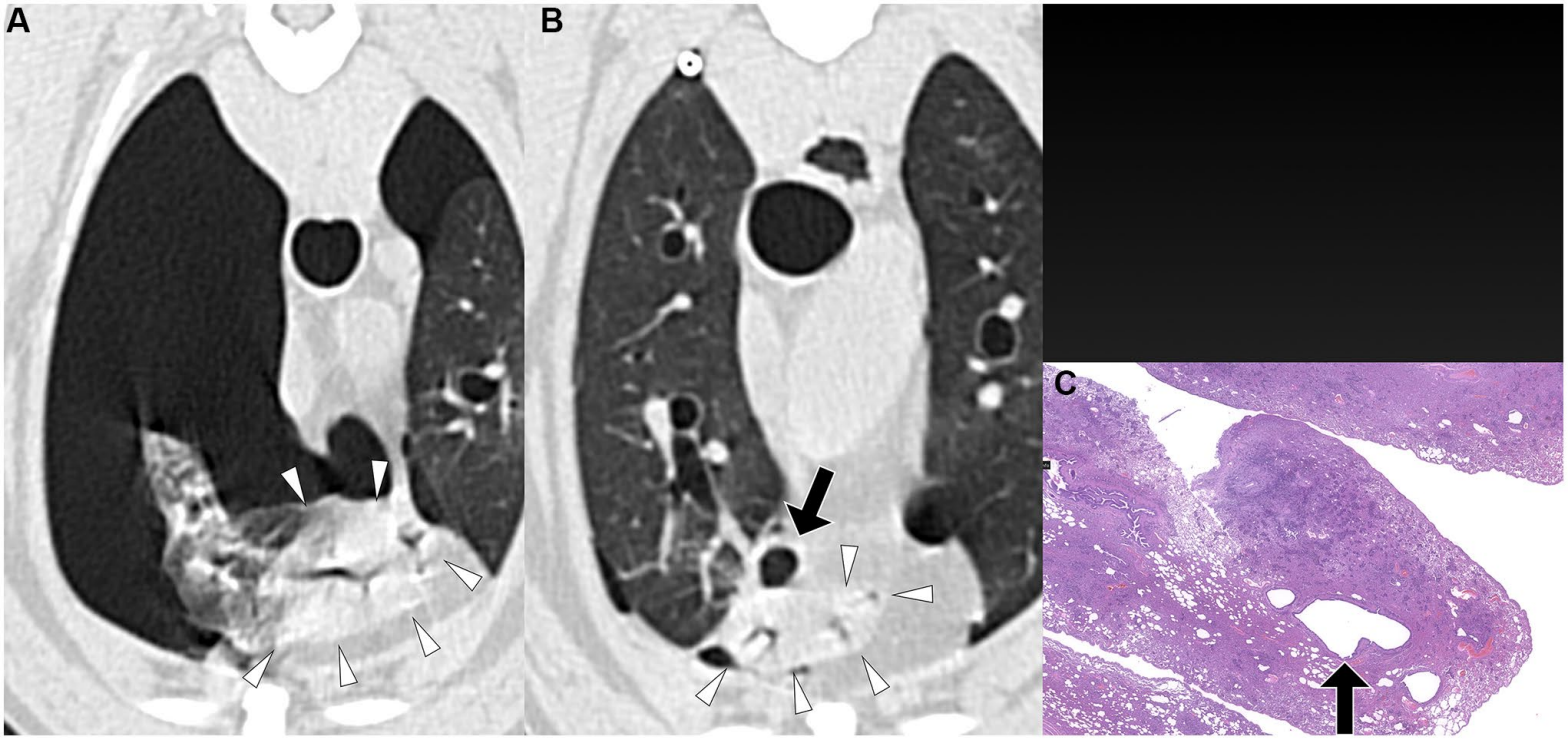
Transverse pre-PPV **(A)** and post-PPV **(B)** CT images of the lungs of a dog with spontaneous pneumothorax using a lung window setting (window plane: −500 HU, window width: 1400 HU). A severe pneumothorax was observed in the left hemithorax, accompanied by marked atelectasis of the left caudal lung lobe (arrowheads) in the pre-PPV CT **(A)**. An air-filled lesion (arrow in **A**) was present along the margin of the left caudal lung lobe on both pre-PPV **(A)** and post-PPV **(B)** CT scans. Post-PPV CT **(B)** showed resolution of lung lobe atelectasis and a slightly enlarged air-filled lesion (arrow in **B**) without additional lesions. CT parameters: 1.388 pitch, 0.5 s rotation time, 120 kVp, 350 mA, and 0.5 mm slice thickness. Histopathological examination of the air-filled lesion (hematoxylin and eosin: HE) **(C)** revealed a bulla (arrow in **C**).

**Figure 2:**
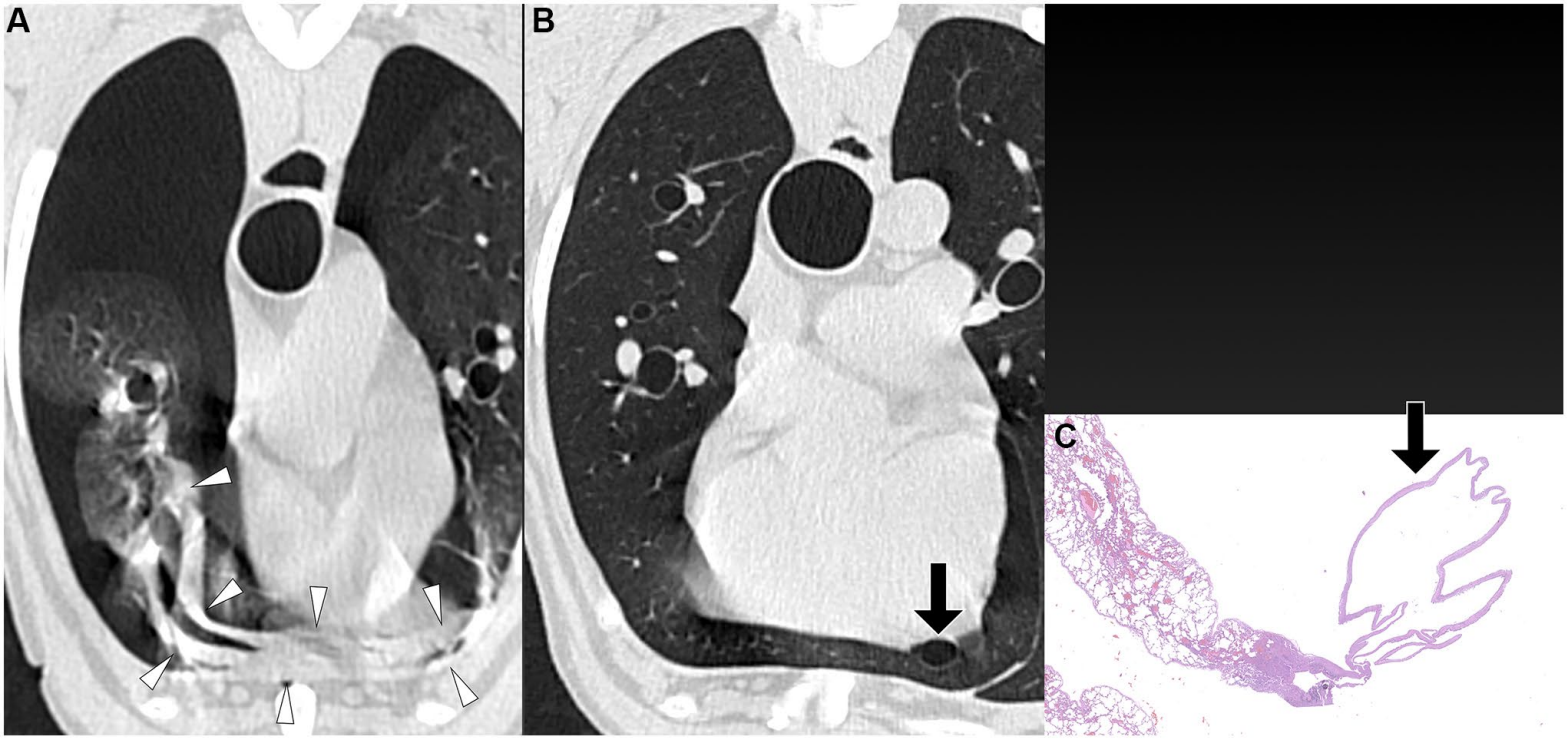
Transverse pre-PPV **(A)** and post-PPV **(B)** CT images of the lungs of a dog with spontaneous pneumothorax using a lung window setting (window plane: −500 HU, window width: 1400 HU). Severe pneumothorax was present in the right hemithorax in pre-PPV CT **(A)**. An air-filled lesion (arrow in **A,B**) was present in the right caudal lung lobe in both pre- **(A)** and post-PPV **(B)** CT. Lung lobe atelectasis was not present. CT parameters: 1.388 pitch, 0.5 s rotation time, 120 kVp, 350 mA, and 0.5 mm slice thickness. Histopathological examination of the air-filled lesion (HE) **(C)** revealed a metastatic sarcoma (arrow in **C**).

**Figure 3:**
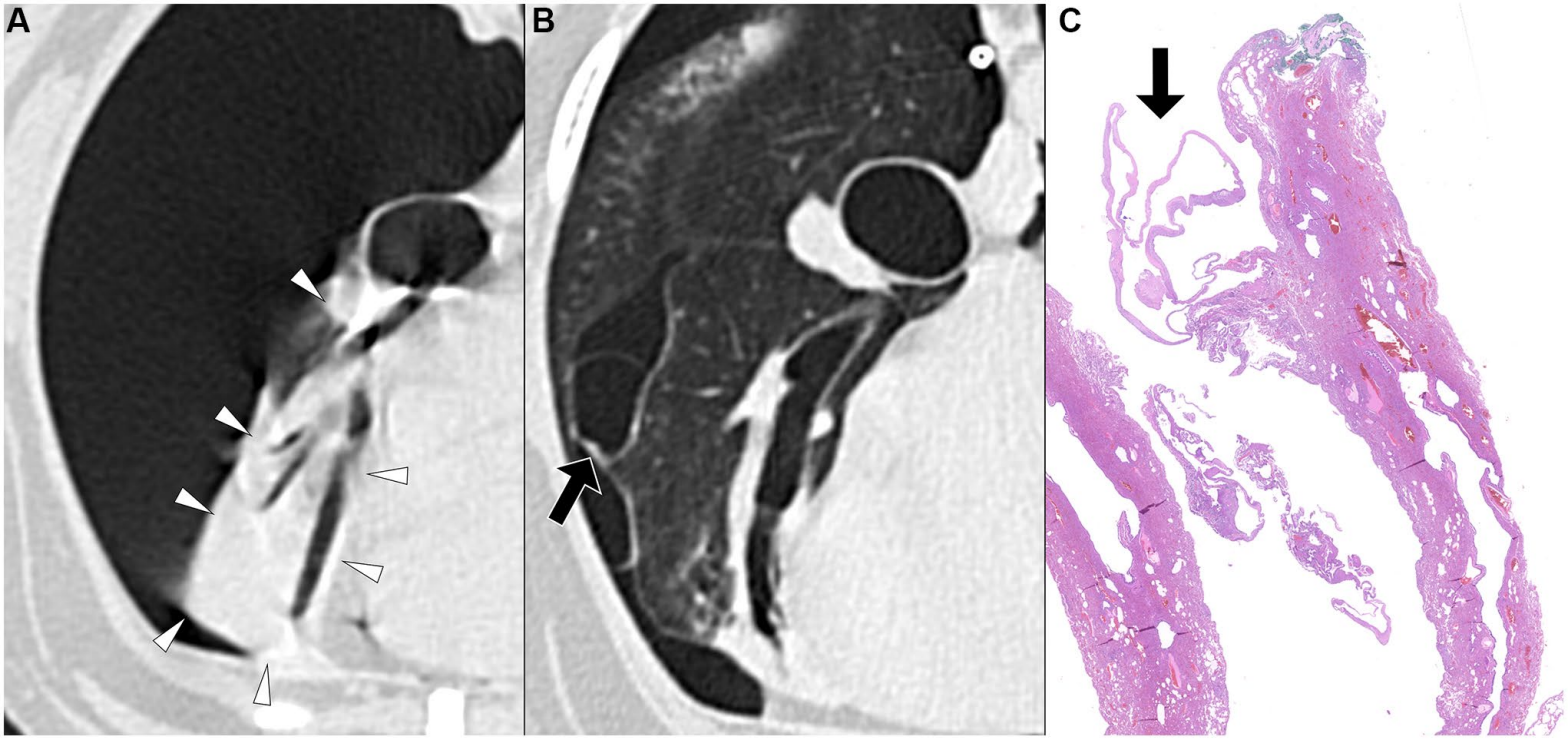
Transverse pre-PPV **(A)** and post-PPV **(B)** CT images of the lungs of a dog with spontaneous pneumothorax using a lung window setting (window plane: −500 HU, window width: 1400 HU). Severe pneumothorax was present in the right hemithorax with severe atelectasis of the right middle lung lobe (arrowheads) in pre-PPV CT **(A)**. There was no visible air-filled lesion in pre-PPV CT. The pneumothorax was improved and lung lobe atelectasis was resolved in post-PPV CT **(B)** with visible air-filled lesion (arrow) along the lateral margin of the right middle lung lobe. CT parameters: 1.388 pitch, 0.5 s rotation time, 120 kVp, 350 mA, and 0.5 mm slice thickness. Histopathological examination of the air-filled lesion (HE) **(C)** revealed a bulla (arrow in **C**).

**Figure 4:**
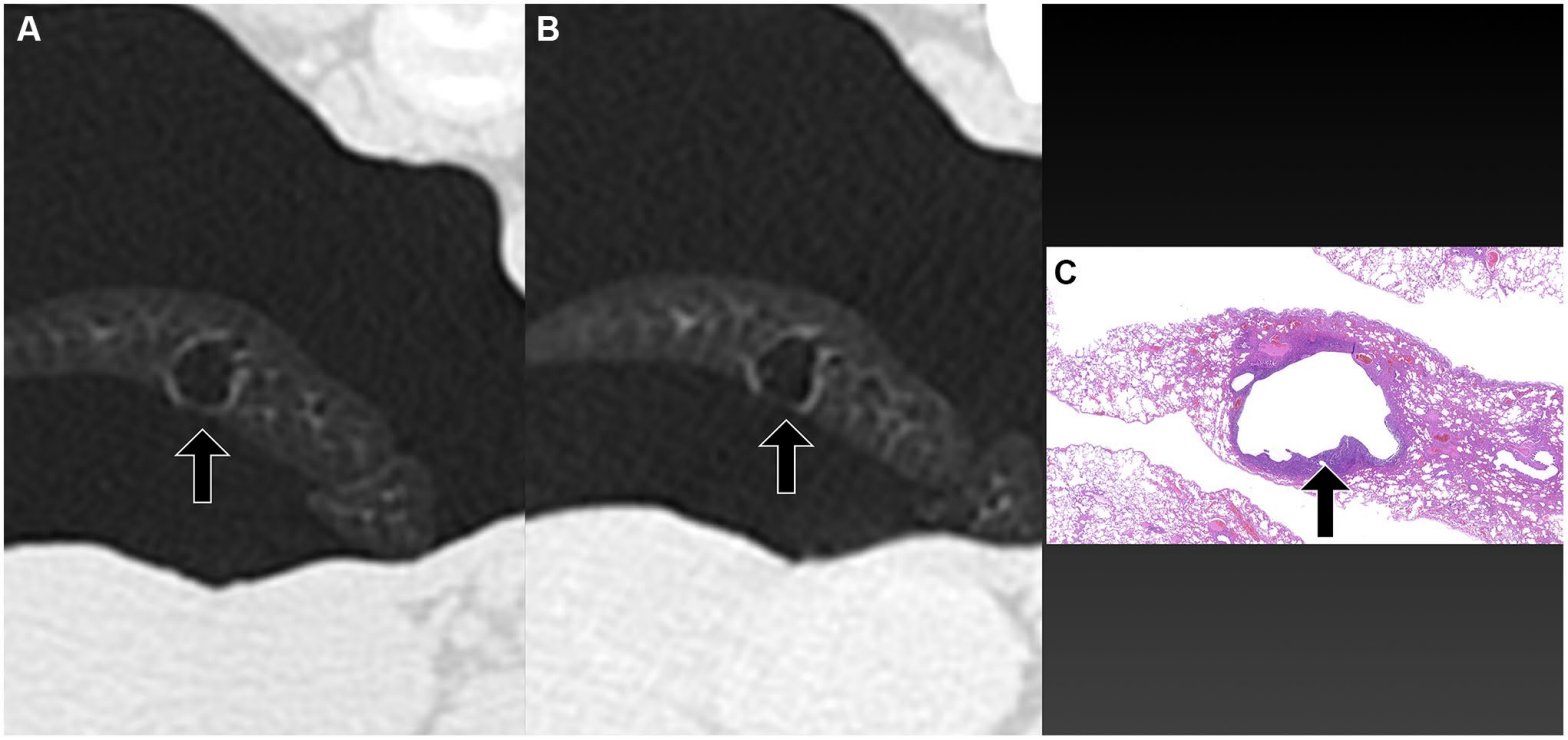
Transverse pre-PPV **(A)** and post-PPV **(B)** CT images of the lungs of a dog with spontaneous pneumothorax using a lung window setting (window plane: −500 HU, window width: 1400 HU). Severe pneumothorax was present in the right hemithorax with severe atelectasis of the right middle lung lobe (arrowheads) in pre-PPV CT **(A)**. There was no visible air-filled lesion in pre-PPV CT. The pneumothorax and lung lobe atelectasis were resolved in post-PPV CT **(B)** with visible air-filled lesion (arrow) along the medial margin of the right middle lung lobe. CT parameters: 1.388 pitch, 0.5 s rotation time, 120 kVp, 350 mA, and 0.5 mm slice thickness. Histopathological examination of the air-filled lesion (HE) **(C)** revealed a bleb (arrow in **C**).

**Figure 5:**
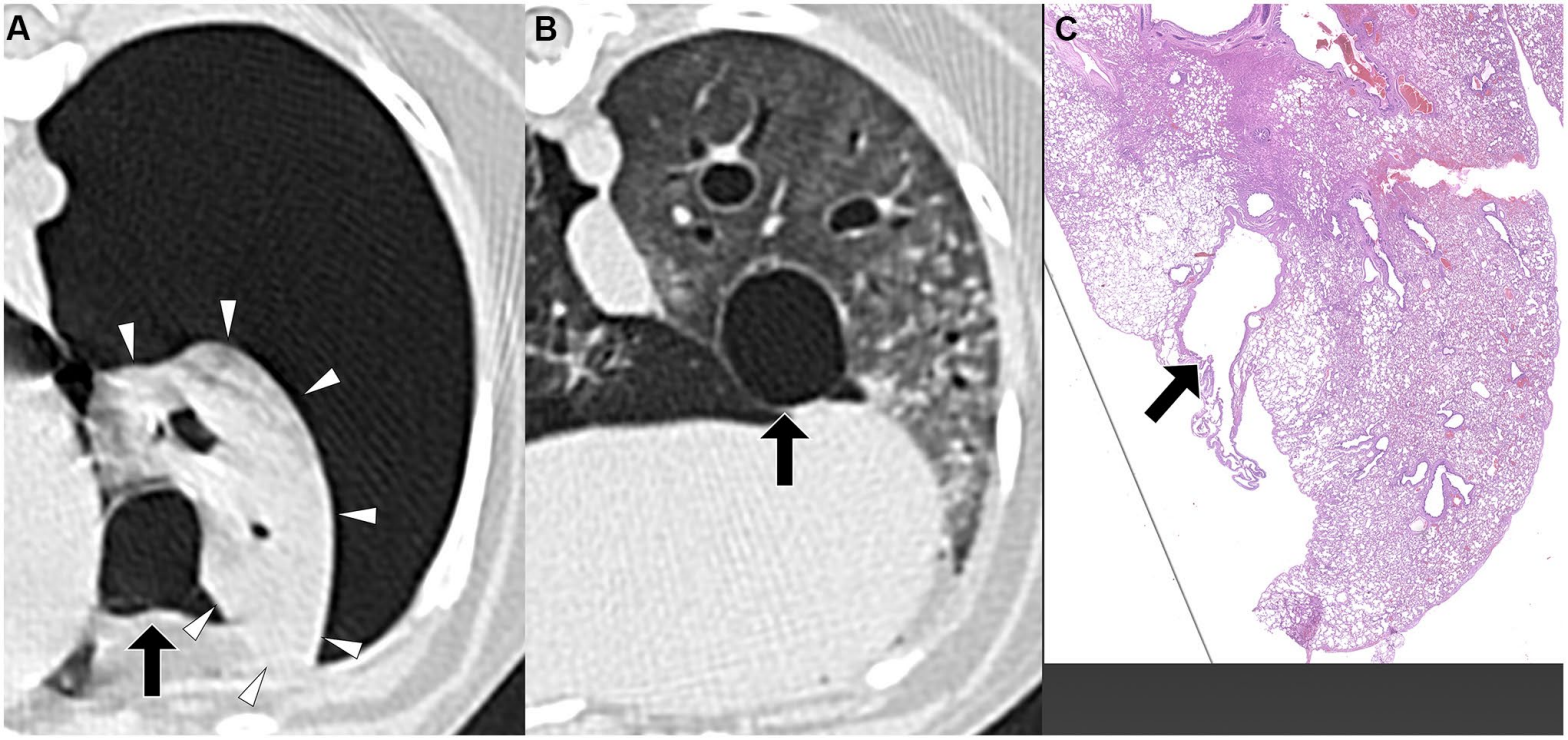
Transverse pre-PPV **(A)** and post-PPV **(B)** CT images of the lungs of a dog with spontaneous pneumothorax using a lung window setting (window plane: −500 HU, window width: 1400 HU). Severe pneumothorax was present in the right hemithorax with moderate atelectasis of the right cranial lung lobe with focal consolidation (arrowheads in **A**) in pre-PPV CT **(A)**. There was no visible air-filled lesion in pre-PPV CT. The pneumothorax and lung lobe atelectasis were resolved in post-PPV CT **(B)**, however, the focal consolidation was unchanged (arrowheads in **B**). An air-filled lesion (arrow) was present adjacent to the aforementioned consolidation in the right cranial lung lobe. CT parameters: 1.388 pitch, 0.5 s rotation time, 120 kVp, 350 mA, and 0.5 mm slice thickness. Histopathological examination of the air-filled lesion (HE) **(C)** revealed bronchiectasis (arrow in **C**).

Air-filled lesions were detected in 5 dogs (5/8; Figures 1A, 2A) and not detected in 3 dogs (Figures 3A–5A) in pre-PPV CT and 8 dogs (8/8) in post-PPV CT (Figures 1B–5B). The number of air-filled lesions detected in post-PPV CT were 1 in 4 dogs, 2 in 2 dogs, and 3 in 2 dogs. All air-filled lesions detected in pre-PPV CT were detected in the same location in post-PPV CT. The mean, median, and range of air-filled lesion diameter were 19.7 mm, 14.7 mm, and 4.3 to 66.1 mm, respectively. Air-filled lesions were present, 2 in the cranial subsegment of the left cranial, 1 in the left caudal, 4 in right cranial, 6 in the right middle, and 1 in the right caudal lung lobes. There was no air-filled lesion detected in the caudal subsegment of the left cranial lung lobe. Eleven air filled lesions were rounded and 3 were irregular in shape. Degrees of pneumothorax in pre-PPV CT was mild in 6, moderate in 4, and severe in 6 pleural cavities, compared to mild in 14, and moderate in 2 pleural cavities in post-PPV CT. None of the dogs showed severe pneumothorax in post-PPV CT.

### 3.4 Assessment of potential risks of PPV

The severity of pneumothorax was assessed using radiographs acquired prior to the CT study and on the following morning. Thoracic radiographs were obtained for all 8 dogs prior to the CT study, and for 7 dogs the following morning. In one dog, a thoracic radiograph was not performed the following morning due to its stable respiratory status, exhibiting a respiratory rate of 20/min. Between the CT studies and the subsequent examinations the following morning, six dogs were maintained in a room with 20%–40% oxygen, including two dogs on active thoracic tube suction. No detectable increase in pneumothorax severity was observed in the 7 dogs with thoracic radiographs obtained both before and after the CT study. The severity of pneumothorax was moderate in 3 and severe in 5 dogs before CT, mild in 5 and moderate in 2 on the following morning. Respiratory rates were recorded for 7 dogs the morning after the CT study, while one dog’s respiratory rate was not documented due to panting.

The mean and median respiratory rates among these 7 dogs were 30.9 and 32/min in 7 dogs, respectively. The volume of air evacuated between the CT study and the following morning was not recorded for 2 dogs due to the use of active suction. In the remaining 6 dogs, the mean volume of evacuated air was 190.5 mL, with individual values of 38, 96, 135, and 874 mL, respectively. Of note, the dog that evacuated 874 mL of gas had a very mild pneumothorax on radiographs obtained the next morning following the CT study.

### 3.5 Surgical confirmation of air-filled lesions

The time interval from CT study to surgery varied between dogs, ranging from a minimum of 1 day to a maximum of 15 days. The specific intervals for each dog were 1 day (three dogs), 3 days (one dog), 4 days (one dog), 5 days (one dog), 7 days (one dog), and 15 days (one dog). Unilateral thoracotomies were performed in all 8 dogs: one in the left hemithorax and seven in the right hemithorax. The decision for the side of thoracotomy was made based on the location of the air-filled lesions observed on CT studies. Of the eight dogs, seven underwent a single lung lobectomy (involving two right cranial lobes, two right middle lobes, one right caudal lobe, one cranial subsegment of the left cranial lobe, and one left caudal lobe), while one dog required lobectomy of two lobes (the right cranial and right middle lobes). In all dogs, air-filled lesions identified during surgery corresponded to the locations detected in the pre-or post-PPV CT. No additional air-filled lesions were found during surgery other than those identified in the CT study. The dogs did not require any special perioperative care, and no recurrence of pneumothorax was observed in any of the dogs postoperatively at the time of writing. Histopathological diagnoses included bullae in 4 dogs, blebs in 2 dogs, bronchiectasis in 1 dog, and metastatic sarcoma in 1 dog, as illustrated in Figures 1C–5C.

## 4 Discussion

In dogs, spontaneous pneumothorax is predominantly attributed to the rupture of air-filled structures, such as bullae or blebs (12–15). Preoperative identification and assessment of these lesions are crucial for surgical planning. CT is regarded as a superior modality for detecting pulmonary air-filled lesions in dogs with spontaneous pneumothorax when compared to thoracic radiography (20). However, the reported detection sensitivities of CT for pulmonary air-filled lesions in dogs with spontaneous pneumothorax vary considerably, ranging from 42.3% to 75% (13, 20). Consequently, the clinical efficacy of CT in detecting air-filled lesions in dogs with spontaneous pneumothorax has been deemed limited (12). In the current study, the initial CT examination without positive pressure ventilation (pre-PPV CT) yielded a detection sensitivity of 62.5% (5/8 dogs) for air-filled lesions, which aligns with previous findings (13, 20).

PPV with a positive end-expiratory pressure of 10 cmH_2_O has been shown to alleviate pulmonary atelectasis in dogs (24), facilitating the evaluation of thoracic CT for various canine diseases. While PPV is known to enhance the visibility of bullae and blebs in humans (25, 26), a study involving dogs with spontaneous pneumothorax reported no improvement in bullae detection with PPV (13, 20). However, the degree of atelectasis following application of PPV was not described in that study (13). In the current study, the sensitivity of pulmonary air-filled lesion detection was improved with the resolution of pulmonary atelectasis by evacuation of pneumothorax and positive pressure ventilation during CT studies. Air-filled lesions were identified in all 8 dogs in post-PPV CT. After evacuating gas from the pleural cavity via a thoracic tube or thoracocentesis, some lung lobes remained atelectatic. In some dogs, a single application of PPV between pre-PPV CT and post-PPV CT, as well as additional PPV for several minutes, was required to completely resolve lung atelectasis. In the current study, multiple air-filled lesions, which were only detected in the post-PPV CT, were located in regions of atelectasis observed in the pre-PPV CT (Figures 3A,B–5A,B). Therefore, it is recommended that PPV be continued in dogs with spontaneous pneumothorax until all areas of atelectasis have resolved.

The potential risks of PPV in dogs with spontaneous pneumothorax include exacerbation of pneumothorax and deterioration of ruptured pulmonary bullae/blebs (25, 26). Despite these risks, prior studies have demonstrated that PPV can be safely managed with thoracic tube placement (13, 20). The day after the CT studies, all dogs, except one that was panting during the physical exam, exhibited normal respiratory rates. Post-CT thoracic radiographs revealed no increased pneumothorax in any of the 7 dogs evaluated. Radiographs were not taken for one dog due to the observation of normal respiration during a physical examination and the absence of gas evacuation from the thoracic tube overnight. It is important to note that the respiratory rates and pneumothorax severity assessed the next morning after the PPV-CT procedure were likely influenced by the pneumothorax evacuation performed after the PPV-CT. Therefore, this study cannot evaluate the risk of PPV in worsening pneumothorax in cases of recurrent spontaneous pneumothorax. However, our findings indicates that PPV is a safe procedure for managing spontaneous pneumothorax in dogs with thoracic tube placement.

Median sternotomy is a commonly used surgical approach to treat spontaneous pneumothorax in dogs (14). However, complication rates associated with median sternotomy have been reported to range from 17% to 78% (27–29). Although accurate detection and localization of air-filled lesions within the pleural cavities are essential, an intercostal thoracotomy might be a more favorable option for lung lobectomy. Studies have demonstrated that dogs undergoing lung lobectomy via intercostal thoracotomy experienced improved short-term outcomes in terms of postoperative pain, oxygenation, and complications compared to median thoracotomy (30). In this study, a lateral thoracotomy was conducted in all dogs, and air-filled lesions were identified and resected based on the findings from the preoperative CT examination. At the time of manuscript preparation, there have been no recurrences of pneumothorax in the 8 dogs studied. A lateral thoracotomy approach may potentially reduce surgical duration and decrease postoperative complications in dogs with spontaneous pneumothorax, particularly when preoperative detection of the air-filled lesion is feasible.

One of the limitations of the present study is the lack of statistical analysis due to the retrospective and cross-sectional design of the study, coupled with a small population of only eight dogs with recurrent spontaneous pneumothorax. This limited sample size hindered the availability of data for substantive statistical comparison. Therefore, we chose a descriptive approach without statistical analysis. Future research with a larger cohort and carefully designed control groups and PPV group without evacuation of pneumothorax may provide true evaluation of PPV itself, leading to more profound insights into the efficacy and safety of the methods studied.

In conclusion, the present study highlights the value of CT imaging combined with evacuation of pneumothorax and PPV for detecting pulmonary air-filled lesions in dogs with spontaneous pneumothorax. The use of evacuation of pneumothorax and PPV until the resolution of atelectasis markedly improved the sensitivity of air-filled lesion detection and facilitated accurate surgical planning. Although PPV carries potential risks, thoracic tube placement ensures a safe procedure for managing spontaneous pneumothorax in dogs.

## Data availability statement

The data analyzed in this study is subject to the following licenses/restrictions: the dataset used for this research was the medical record from the veterinary hospital and will be shared if there is appropriate request. Requests to access these datasets should be directed to mmuraka@purdue.edu.

## Ethics statement

Ethical approval was not required for the studies involving animals in accordance with the local legislation and institutional requirements because this study was performed retrospectively acquiring data from medical record, and there was no prospective involvement of animals in the study. Written informed consent was obtained from the owners for the participation of their animals in this study.

## Author contributions

AT: Conceptualization, Data curation, Formal analysis, Investigation, Methodology, Resources, Visualization, Writing – original draft. CF: Formal analysis, Investigation, Methodology, Supervision, Validation, Writing – review & editing. YK: Data curation, Formal analysis, Investigation, Writing – review & editing. MM: Conceptualization, Formal analysis, Investigation, Methodology, Project administration, Resources, Supervision, Validation, Visualization, Writing – original draft, Writing – review & editing.
